# Supplementary figures and images for: Cerebro-spinal flow pattern in the cervical subarachnoid space of healthy volunteers: Influence of the spinal cord morphology
Source: PLoS One. 2024 Aug 26;19(8):e0290927. doi: 10.1371/journal.pone.0290927 (PMC11346662; doi:10.1371/journal.pone.0290927)

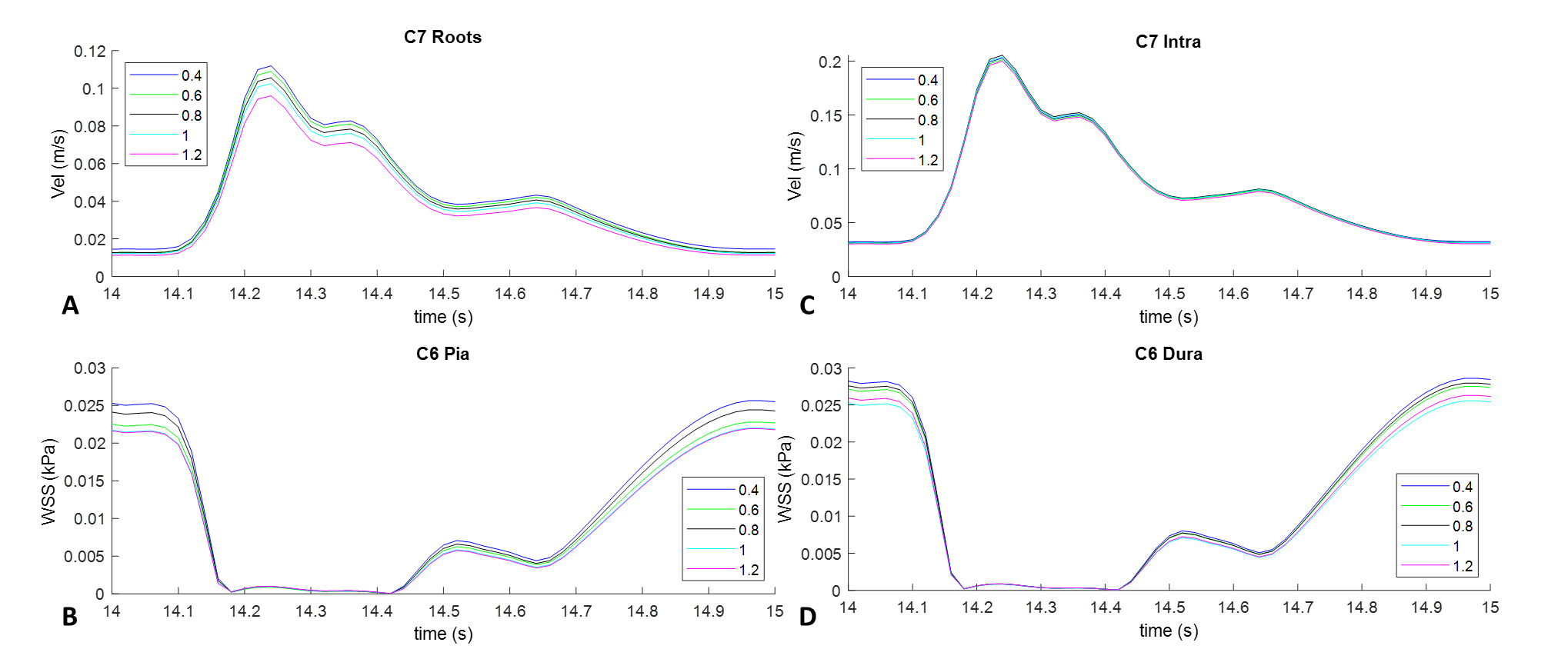

Supplement: S1 Fig — Segmentation, mesh, mesh refinement, layer mesh size (1.2–A, 1 -B, 0.6-C), pipes addition (D). (PNG) [file pone.0290927.s001.png]

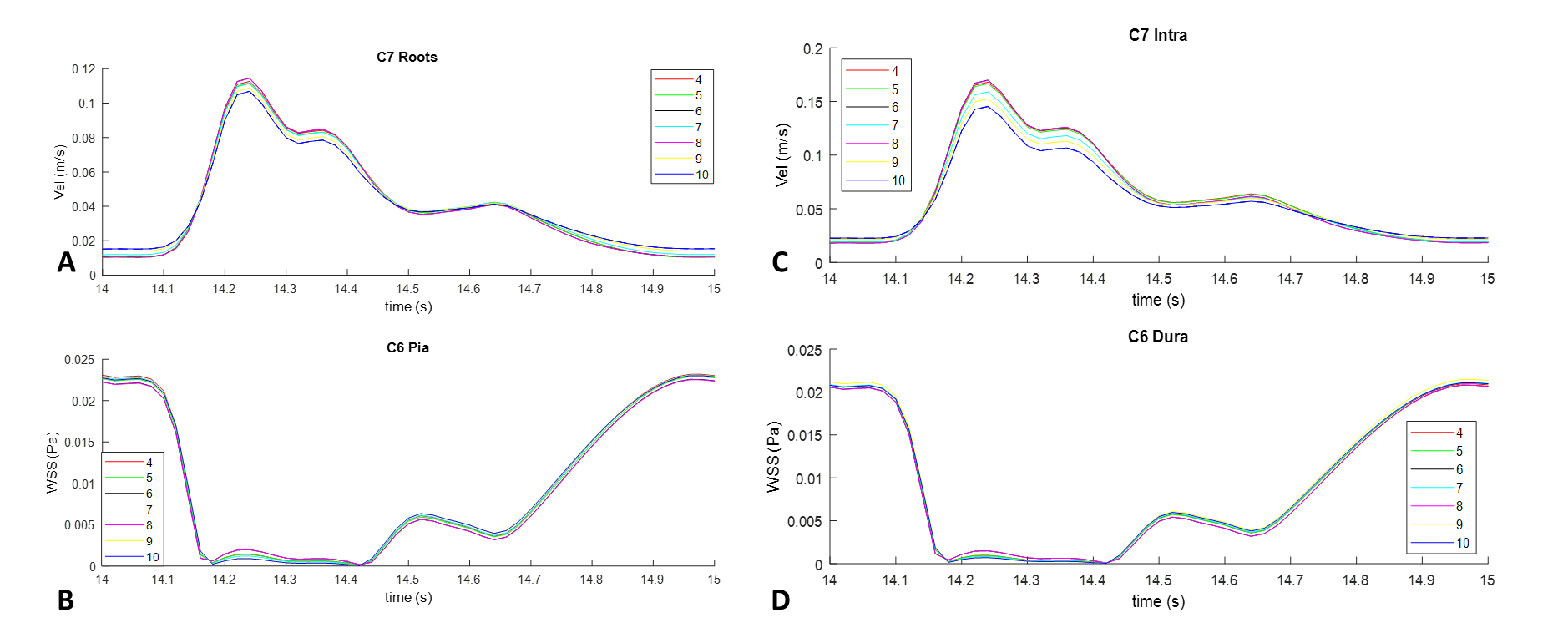

Supplement: S2 Fig — Velocity and WSS profile at C7 and C6 locations with boundary layers number varying from 4 to 10 layers of 0.05mm. (PNG) [file pone.0290927.s002.png]

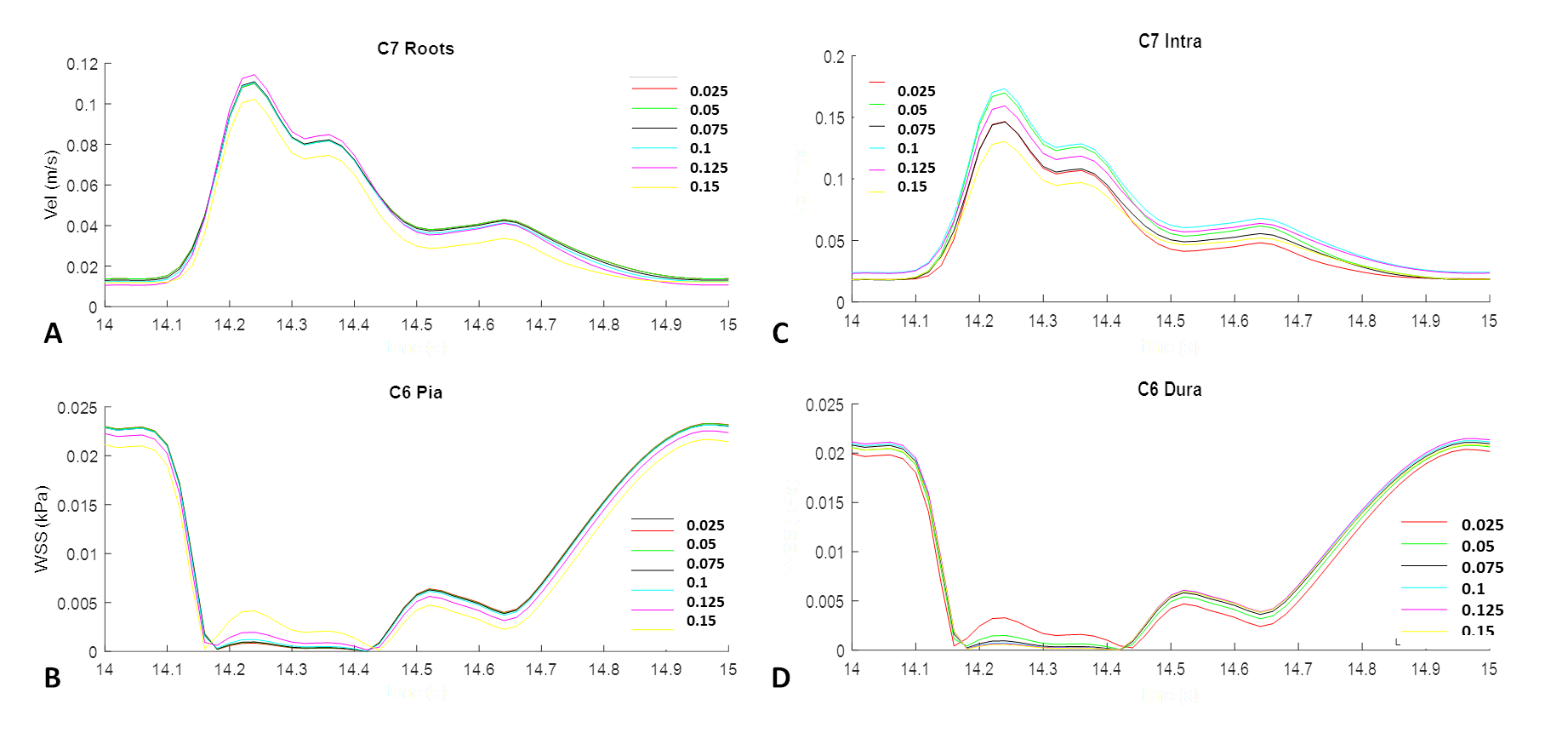

Supplement: S3 Fig — Velocity and WSS profile at C7 and C6 locations with the size of boundary layers varying from 0.025 to 0.15mm. (PNG) [file pone.0290927.s003.png]

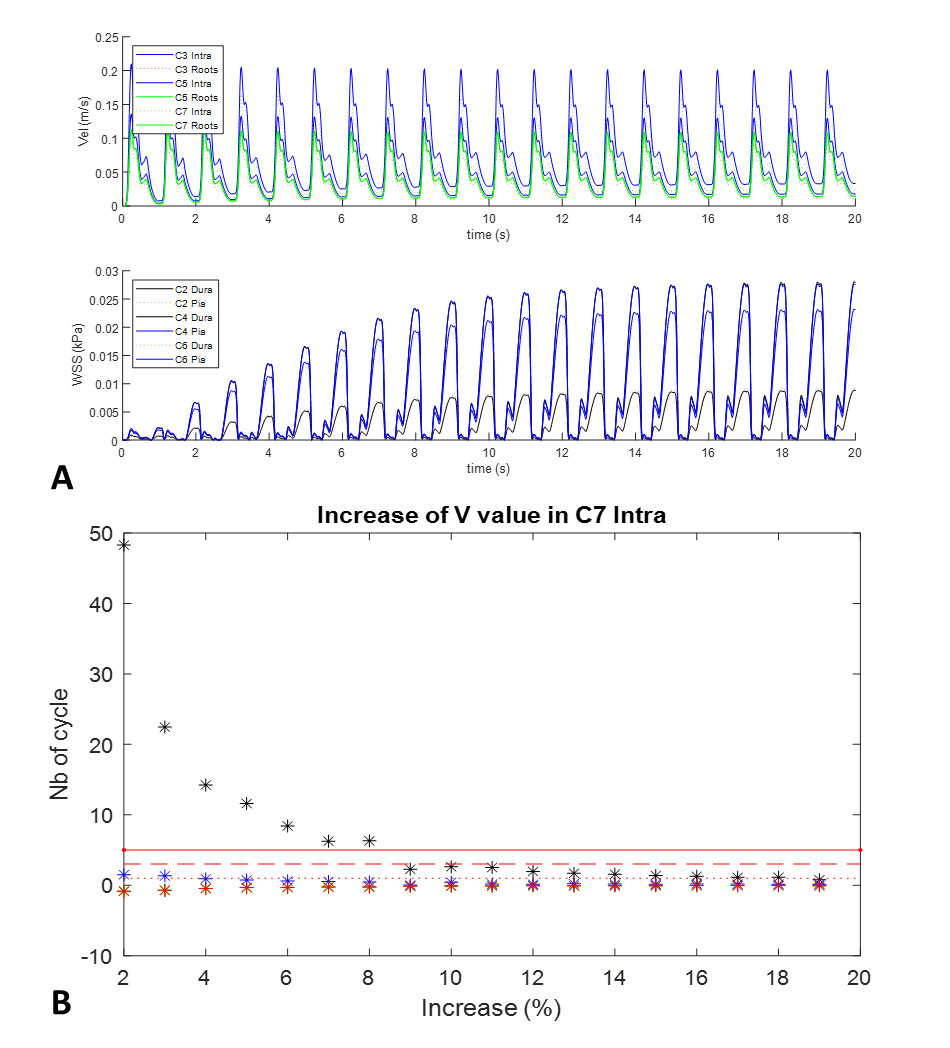

Supplement: S4 Fig — Velocity and WSS profile at different locations for 20 cycles. (PNG) [file pone.0290927.s004.png]

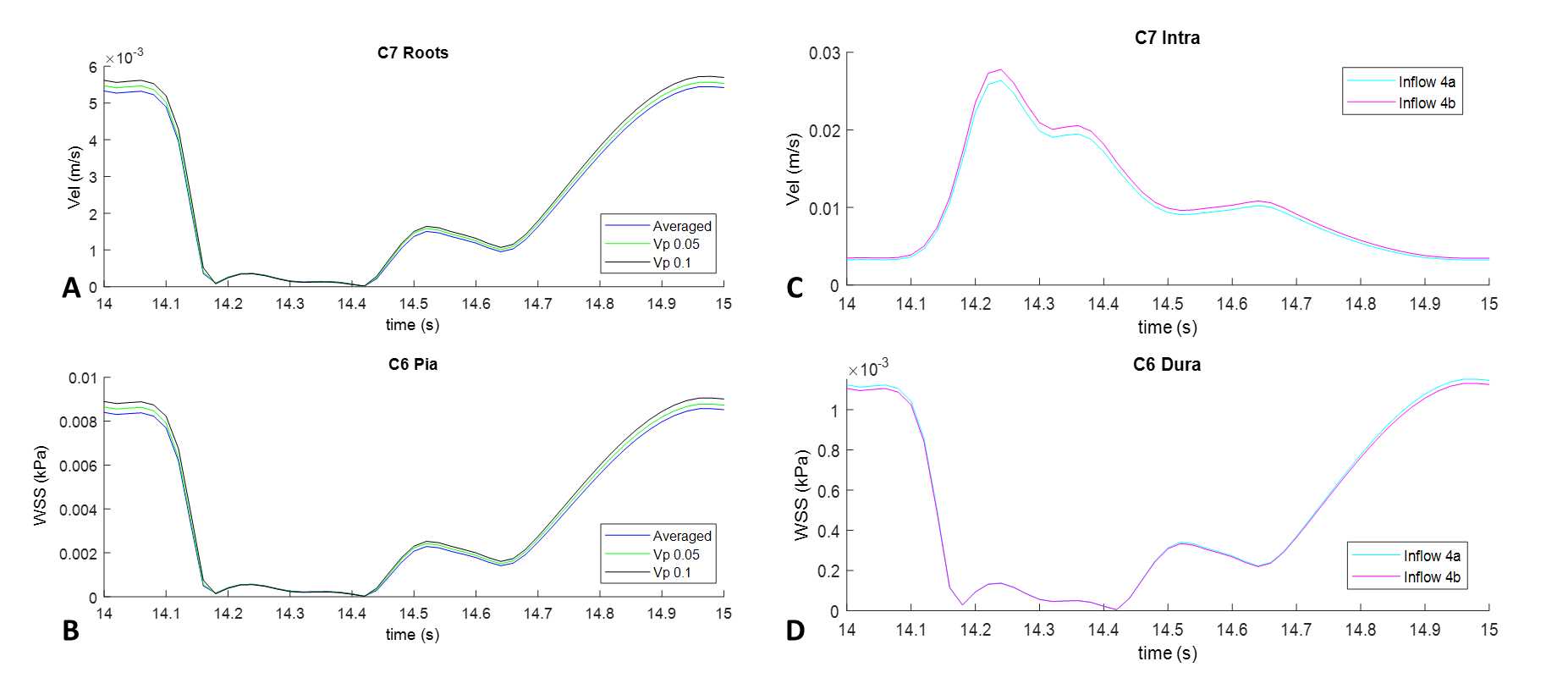

Supplement: S5 Fig — (PNG) [file pone.0290927.s005.png]
