# Supplementary material for: Cerebro-spinal flow pattern in the cervical subarachnoid space of healthy volunteers: Influence of the spinal cord morphology
Source: PLoS One. 2024 Aug 26;19(8):e0290927. doi: 10.1371/journal.pone.0290927 (PMC11346662; doi:10.1371/journal.pone.0290927)
Supplement: S2 File — (DOCX) [file pone.0290927.s007.docx]

Supplementary data - Sensitivity analysis results

*Mesh size*

The velocity values become stable starting from the 0.6 mesh size. When comparing mean values of velocity and WSS for the 0.6 mesh with the ones for the 0.4 and 0.8 mesh, the differences are inferior to 3% and 5% respectively ( see Supplementary data Figure 2).

*Number et size of layers*

When observing the convergence of the velocity values along the cervical subarachnoid canal, the values became stable between the sixth and eight layers. 6 layers were created near the walls as mean velocity values were inferior to 3%. Size of the layer was chosen to be 0.05 (y+ values independently of the layer size are inferior to 6) ( see Supplementary data Figure 3 and 3).

*Number of cycles*

When considering the mean values of velocity and WSS, the percentage of these values change between the fifteenth and the sixteenth cycles were respectively 0.3% and 0.5% of the fifteenth cycle values (see Supplementary data Figure 4).

*Time-step analysis*

The changes in the computation time reflect greatly on WSS values while no significant changes were found in velocity values (differences between mean velocity values of the 3 time-steps studied inferior to 2% while the two by two WSS differences superior to 9.8% in all case). Thus, the smaller time step was chosen (20ms).

*Types of boundary conditions*

As expected, the constant component to the velocity is increasing the WSS (0.20; 0.23; 0.29 Pa). However, such increase is non-linear. Difference between the two parted velocity profile were found negligible (less than 1% of peak velocity and WSS, see Supplementary data Figure 5).
