# Supplementary material for: Cerebro-spinal flow pattern in the cervical subarachnoid space of healthy volunteers: Influence of the spinal cord morphology
Source: PLoS One. 2024 Aug 26;19(8):e0290927. doi: 10.1371/journal.pone.0290927 (PMC11346662; doi:10.1371/journal.pone.0290927)
Supplement: Attachment — Submitted filename: ReplyToEditorsComments_131023.docx [file pone.0290927.s008.docx]

**Journal : PLOS-ONE**

**TITLE:**

Cerebro-spinal Flow Pattern in the Cervical Subarachnoid Space of Healthy Volunteers: Influence of the Spinal Cord morphology

**NB:** PONE-D-23-26218

**OBJECTIVES: Responses to Reviewers’ comments**

*C1 - Please review the translation of the ethical clearance provided with the submission. Since the study has incorporated humans as subjects, there has to be a study specific ethical approval which seems unclear on reviewing the submission. The authors are requested to revisit the submission files and address the issue before it can be sent to the reviewers for further processing.*

Dear Editor,

We are now provided a revised version of the translation of the ethical clearance. However, we would like to underline that the data have been previously published and that all the ethical approval were granted as we have been demonstrated with the provision of the ethical letter and its translation. Moreover, this study uses only 3D subarachnoidal canal segmentation to perform a computational fluid analysis within the subarachnoidal canal. As such and considering the fact that the initial data are already published and validated in term of ethics in [1]*.*

1.	Sudres P, Evin M, Arnoux P-J, Callot V. Cervical canal morphology: Effects of neck flexion in normal condition - New elements for biomechanical simulations and surgical management. Spine. 2020. doi:10.1097/BRS.0000000000003496

C2 - *We note that Figure 1, 2B, 2D, 2E, 3B, 3D, 4, S1 in your submission contain copyrighted images. All PLOS content is published under the Creative Commons Attribution License (CC BY 4.0), which means that the manuscript, images, and Supporting Information files will be freely available online, and any third party is permitted to access, download, copy, distribute, and use these materials in any way, even commercially, with proper attribution. For more information, see our copyright guidelines:* [*http://journals.plos.org/plosone/s/licenses-and-copyright*](http://journals.plos.org/plosone/s/licenses-and-copyright)*.*
 
As previously mentioned in exchange with the editor, all the figures were created by the authors and are not published elsewhere. As such, no copyright is requested.

We sincerely hope that this new version of the translation will be suitable for you to accept transfer for review and would like to mentioned that considering the delay occasioned by this issue we would appreciate a quick reply regarding this manuscript.

Best regards,

Morgane EVIN
