# Supplementary material for: Cerebro-spinal flow pattern in the cervical subarachnoid space of healthy volunteers: Influence of the spinal cord morphology
Source: PLoS One. 2024 Aug 26;19(8):e0290927. doi: 10.1371/journal.pone.0290927 (PMC11346662; doi:10.1371/journal.pone.0290927)
Supplement: Attachment — Submitted filename: ReplyToEditorsComments_120224.docx [file pone.0290927.s009.docx]

**Journal : PLOS-ONE**

**TITLE:**

Cerebro-spinal Flow Pattern in the Cervical Subarachnoid Space of Healthy Volunteers: Influence of the Spinal Cord morphology

**NB:** PONE-D-23-26218

**OBJECTIVES: Responses to Reviewers’ comments**

Reviewer #1: I thank the editor for providing me the chance to share my review insights on this professionally made study. though the study focuses on the craniocervical junction, I would like-however- to inquire regarding the wall shear stress and compression ratio in the 4th ventricular through the central canal as it has marked implications regarding the pathologies of CNS and spinal cord e.g. the specific predilection of Alcohol intoxication on midline inferior vermis.

We thank the reviewer for his/her comment and would like to underline that the study focuses on the cervical spine rather than cranio-cervical junction which will be furtherly study in the next work. Indeed, both the morphology and the fluid dynamics in the craniocervical junction need to be fully studied and was behind the scope of this study. Additionally, the study of the fluid dynamics between 4^th^ ventricle and central canal could also be addressed using analytical model rather than computational fluid dynamic. The present work is a step forward more knowledge on the cerebro-spinal fluid dynamic in healthy cervical spine and brain before addressing pathological context such as Chiari. It is the first to describe correlation between morphology (compression ratio) and flow pattern. We truly hope that based on the presented results, this work will be accepted for publication.

*Reviewer #2: Thank you for the opportunity to review this very interesting paper regarding the cerebro-spinal flow Pattern in the cervical subarachnoid space of healthy volunteers.*

We thank the reviewer for his/her helpful comment and hope to have addressed all and have improve the manuscript.

*Here are my specific comments:*

1. *Abstract- too many abbreviations not commonly known- need to write them in full.*

All abbreviations have been removed from the abstract.

*a. What is the significance of this findings in real world.*

The abstract has been modified in order to highlight the need for cerebro-spinal flow quantification in clinical practice as well as the modification of the cerebro-spinal flow pattern depending on the morphology of the canal.

*b. As the audience comprises of neurologist, neurosurgeons and other neuroscientists, the language used should be tailored to them in an understandable fashion.*

This has been modified through the abstract.

*2. Introduction – a bit long. Can be shortened to deliver the same meaning.*

The introduction has been reduced and clarified.

*3. Please add in the discussion section what could be the potential clinical application of this research.*

A final paragraph on potential clinical application as been added to the discussion.
